# Who Sits Where? Infrastructure-Free In-Vehicle Cooperative Positioning via Smartphones

**DOI:** 10.3390/s140711605

**Published:** 2014-06-30

**Authors:** Zongjian He, Jiannong Cao, Xuefeng Liu, Shaojie Tang

**Affiliations:** 1 Department of Computing, The Hong Kong Polytechnic University, Hong Kong, China; E-Mails: csjcao@comp.polyu.edu.hk (J.C.); csxfliu@comp.polyu.edu.hk (X.L.); 2 Department of Computer and Information Science, Temple University, Philadelphia, PA 19122, USA; E-Mail: tangshaojie@gmail.com

**Keywords:** in-vehicle positioning, smartphone sensing, opportunistic sensing, signal processing

## Abstract

Seat-level positioning of a smartphone in a vehicle can provide a fine-grained context for many interesting in-vehicle applications, including driver distraction prevention, driving behavior estimation, in-vehicle services customization, *etc.* However, most of the existing work on in-vehicle positioning relies on special infrastructures, such as the stereo, cigarette lighter adapter or OBD (on-board diagnostic) adapter. In this work, we propose iLoc, an infrastructure-free, in-vehicle, cooperative positioning system via smartphones. iLoc does not require any extra devices and uses only embedded sensors in smartphones to determine the phones' seat-level locations in a car. In iLoc, in-vehicle smartphones automatically collect data during certain kinds of events and cooperatively determine the relative left/right and front/back locations. In addition, iLoc is tolerant to noisy data and possible sensor errors. We evaluate the performance of iLoc using experiments conducted in real driving scenarios. Results show that the positioning accuracy can reach 90% in the majority of cases and around 70% even in the worst-cases.

## 1. Introduction

Recent years have witnessed a dramatic increase in smartphone-based in-vehicle applications for safety [1] and entertainment [2] purposes. In these applications, a smartphone's in-vehicle seat-level position usually serves as an important context. For example, a driver distraction prevention application can automatically route incoming calls to voicemail or prevent texting while driving. It only needs to run on the driver's phone instead of the passengers'. Another interesting application is in-vehicle services customization, which can give passengers a seamless entertainment experience, allowing for selected media sources to be handed off from the mobile device to the corresponding headrest monitor. Information, like “who sits where”, is also necessary for handing off the media previously played in the passengers' smartphones to the proper headrest monitors.

Different approaches have been developed to obtain a phone's position information. The driver's manual input has been used by Android, Windows Phone and many other applications to activate a “driving mode” before driving. However, this approach is not convenient. Another interesting approach is to utilize in-vehicle infrastructure, such as a Bluetooth stereo [3,4], a cigarette lighter [5] or the OBD (on-board diagnostic) system [5], as a beacon to localize the phones. By communicating with infrastructures, the phone positions can be identified automatically. However, these infrastructures are not always available for all vehicles.

In this paper, we propose a novel solution, named iLoc, to determine the seat-level (front/back, left/right) positions of smartphones using their embedded sensors only. The key idea of iLoc is to analyze the difference in the phones' acceleration at different positions when some particular events occur. Specifically: (1) when a vehicle is turning, the difference in the centripetal accelerations measured at different smartphones is utilized to determine which one is to the left/right of another; and (2) when the car is passing uneven road surfaces, the difference in the vertical accelerations measured at smartphones is utilized to determine the front/back positions. Compared with existing work, iLoc has the following advantages:

- Infrastructure-free: iLoc does not rely on any dedicated devices in vehicles.
- Robustness: iLoc can correctly identify the positions regardless of the placement and orientation of the phones in a noisy environment.
- Energy efficiency: iLoc samples data only when particular events occur and avoids the usage of high power consumption smartphone sensors, like GPS and camera.

However, implementing iLoc entails substantial challenges: (1) Collected data contain lots of noise. It is very difficult, if not impossible, to determine the positions of smartphones directly using the raw data; (2) Without infrastructure, the phones can only utilize limited information from each other in an *ad hoc* way. This significantly increases the difficulty in designing the positioning algorithms; (3) To compare the acceleration data from different phones, the phones must be strictly synchronized. Exchanging beacon signals is not practical in smartphones, due to the uncertain wireless delay. In addition, sensor sampling jitters and clock crystals errors can also cause uncertain delay.

In summary, we propose a seat-level in-vehicle positioning system with the strengths of being infrastructure-free, robust and energy efficient. The contributions are as follows:

- We design an event-triggered positioning method. The method detects several predefined events, and the positioning process, including data collection, transmission and comparison, is triggered by events. It considerably reduces the energy consumption and enables the system to re-position if users change the phone's position, e.g., picking up the phone from one pocket, checking the time, and then dropping it in another pocket.
- We develop cooperative positioning algorithms for infrastructure-free sensing. More specifically, for left/right identification, we develop synchronized sensing and amplitude calibration algorithms to mitigate the noise. For front/back identification, we develop sliding-SD to differentiate the signal peaks.
- We implement iLoc on Android phones and evaluate its performance in real city driving scenarios. Evaluation results show that the positioning accuracy can reach 90% in the majority of cases and around 70% in even the worst scenarios.

The rest of this paper is organized as follows. In Section 2, we describe related work and address the differences. The system overview is presented in Section 3. From Section 4 to Section 7, we describe some key components in the system. Section 8 introduces the extensive evaluations and field test. In Section 9, we discuss some possible aspects for future improvement and conclude the paper in Section 10.

## 2. Related Work

### 2.1. Seat-Level In-Vehicle Positioning

Active research has been conducted in smartphone-based seat level in-vehicle positioning. Yang *et al.* [3,4] proposed an interesting solution to determine the in-vehicle position of smartphones automatically. In this approach, smartphones first control, via Bluetooth, the onboard stereo to send beep signals from different corners of the vehicle and then determine locations based on the arrival time of the beeps received at the microphones. However, this method assumes that the vehicle has an onboard Bluetooth stereo at four corners, which only holds for high-end vehicles. Meanwhile, sound waves can be easily affected by other in-vehicle sound, like engine noise. Yet, another approach, “this is me” [6], identifies a phone's position based on voice patterns (a.k.a. the voiceprint) of passengers to provide different multimedia services for passengers. However, this approach requires hi-fidelity directional microphones installed in front of each front-seat and, therefore, is infrastructure-heavy. In addition, each passenger needs to provide voice data to train the system, which is not very practical in real applications. Differently from the above approaches, iLoc relies on neither external infrastructures nor a pre-sampled database.

Chu *et al.* [7] proposed an infrastructure-free solution to differentiate driver and passengers by tracking their micro-movements during events, like vehicle boarding and seat belt fastening, using smartphone sensors. Unlike iLoc, The positioning is completed before driving rather than during driving. Thus, it is unable to correct positioning errors after driving started and can not handle phone position change. In addition, its performance can potentially be affected by different types of cars, where the mobile phone is placed and various types of human factors.

Toward the most related work in in-vehicle positioning, Wang *et al.* [5] proposed an accelerometer-based solution that shares the same idea as iLoc by comparing the centripetal acceleration during vehicle turn for left/right identification and discussed front/back identification in their previous work [4]. iLoc differs from their work in the following aspects: (1) iLoc does not use a cigarette lighter or OBD II as the external reference, but only uses two phones (having two phones in a vehicle is more practical and feasible than the presence of infrastructure). Hence, the beacon-based positioning cannot be directly applied to our scenario. The algorithms in this paper are dedicatedly designed for the infrastructure-free scenario. (2) By using a cigarette lighter or OBD II interface, the system can keep on sensing without considering energy efficiency, since the ports are directly connected to vehicle battery. In iLoc, we implemented event-triggered positioning to save energy. (3) Yang *et al.* [4] discussed a possible front/back identification approach by identifying two “spike-like acceleration peaks” when the vehicle passes uneven road surfaces. However, according to our real experiment, such “two-peak pattern” is not always obvious. To tackle this problem, we develop sliding-SD based on cooperative positioning.

### 2.2. Situational Aware Study

Sensors in smartphones have also been widely used in many situationally aware applications. iLoc also has the potential to be applied to develop such applications by providing a fine-grained in-vehicle context. We survey smartphone-based vehicular situationally aware applications in this section.

Reddy *et al.* [8] proposed a method of using smartphones to detect users' transportation mode, such as stationary, running or taking vehicles. Stenneth *et al.* [9] improved the results by taking GIS information into consideration. ActraMon [10] is a framework providing information about where people and vehicles are located, how they move in urban areas and which activities they do at places in certain patterns. Bojja *et al.* [11] developed a 3D indoor localization solution that can be used inside parking lots. We believe that, by combining iLoc with the aforementioned situationally aware techniques, many interesting applications can become feasible.

## 3. System Overview

**Figure 1.**
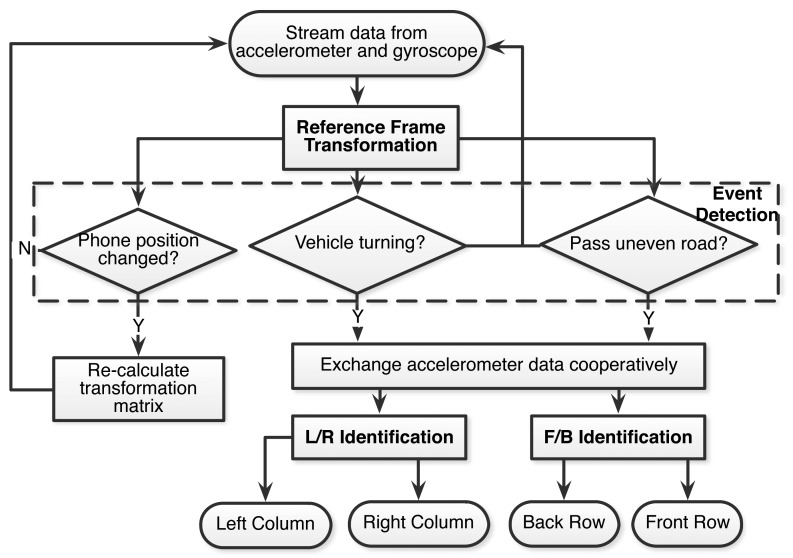
System overview and flow chart of iLoc.

iLoc includes several key components, including reference frame transformation, event detection and left/right, front/back identification. Figure 1 depicts the flow chart of iLoc. First, after the system is initialized, all smartphones start to continuously collect stream data from their onboard accelerometers and gyroscopes. Then, the collected data are transformed from the phone frame to the vehicle frame based on the transformation matrix calculated from the phone's orientation. The event detection module then keeps detecting whether some predefined events have occurred. Once an event is detected, the corresponding algorithm is invoked to detect the phones location. During this process, if the phones' position or orientation is changed by the users, iLoc will re-calculate the transformation matrix and repeat the above positioning procedure. iLoc terminates after identifying all phones' seat-level positions.

## 4. Reference Frame Transformation

**Figure 2.**
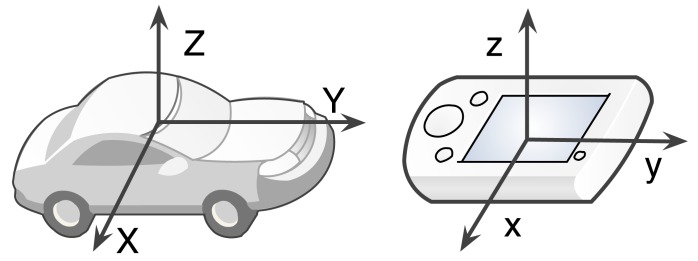
The reference frames. (**left**) vehicle frame; (**right**) phone frame.

The main purpose of reference frame transformation is to handle the different orientations caused by arbitrary phone placement, like inside a pocket, a handbag, on the car console or even in the hand. How to eliminate the effect on positioning accuracy must be carefully addressed. The basic idea is to transform the sensor readings from the device-dependent phone frame to the device independent vehicle frame. The corresponding reference frames are depicted in Figure 2. We use (*X, Y, Z*) and (*x, y, z*) to denote the vehicle frame and phone frame, respectively. Some researches use fixed phone placement (e.g., mounted on the windshield) to avoid reference frame transformation [12–14]. However, considering the widespread arbitrary phone placement scenario, the transformation is a necessary step.

To determine the transformation matrix between the two reference frames, two orthogonal vectors and their projections are required. The two vectors we choose are gravity acceleration and deceleration during translational movement (driving in a straight line). A similar approach in also adopted in Nericell [15]. Nericell needs to collect data when the vehicle is driving on a straight line for calculating the transformation matrix. Unlike Nericell, we replace energy-consuming GPS with a gyroscope to determine vehicle deceleration during straight line driving. It is detected when the root sum square of the accelerometer reading $∥\vec{a}∥=\sqrt{a_{x}^{2}+a_{y}^{2}+a_{z}^{2}}\geq \eta$ and the root sum square of the gyroscope reading $∥\vec{\omega }∥=\sqrt{\omega_{x}^{2}+\omega_{y}^{2}+\omega_{z}^{2}}\leq \psi$ where *η* and *ψ* are two predefined thresholds. In this work, we use *η* = 0.11 *g* and *ψ* = 0.3. Since this is not a main contribution of this work, the user may refer to the original Nericell paper [15] if interested.

## 5. Event Detection

The event detection module analyzes the transformed sensor data to determine whether the predefined events occur and triggers corresponding positioning algorithms. Specifically, three events are defined: (1) when the vehicle turns, the left/right positioning algorithm is activated; (2) when the vehicle passes an uneven surface, the front/back positioning algorithm is triggered; and (3) when the phone position changes, the transformation matrix from the phone frame to the vehicle frame is re-calculated. It should be noted that since the event detection algorithm runs continuously, the algorithm itself must be light-weight and highly efficient.

**Figure 3.**
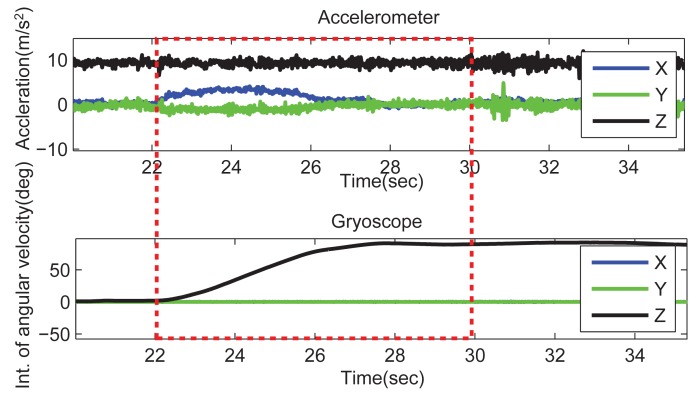
Sensor data during a “vehicle turn” event.

When a vehicle turns, both the car and phones have an angular velocity around the *z*-axis in the vehicle frame, which can be detected by the phone's gyroscope. We can obtain the degree that the car has rotated using cumulative trapezoidal numerical integration on the angular velocity. Then, a sliding window is used to determine the event. When the integrated degree is larger than a predefined angle (say, 3π/8), the event detection module determines that a “vehicle turn” event has occurred. Figure 3 depicts the integration of gyroscope data and the raw accelerometer data when a vehicle turns at a road intersection. It can also be found that given a certain threshold, the smaller the window size, the less the number of events that are detected. The effect of different sliding window sizes will be evaluated in our experiment.

When a vehicle passes an uneven surface, such as bumps or potholes, there will be accelerations along the *z*-axis. The acceleration is caused by the vertical deflection of the front and back wheels. Existing works [13,16] have designed algorithms to detect potholes and bumps. We use a similar acceleration threshold on the *z*-axis to determine the occurrence of the event. If the threshold is satisfied, a “vehicle passes uneven surface” event is confirmed.

When a phone's position is changed by users. The gyroscope can sense the circular movement around an arbitrary axes. Differently, when a vehicle runs normally, the gyroscope reading should mainly reflect the vertical orientation. Hence, We calculate the rotation angle of all three axes, and if circular movement around *x* or *y* exceeds a predefined value, we conclude that the phone's position is changed and that the transformation matrix needs to be recalculated.

## 6. Left/Right Identification

**Figure 4.**
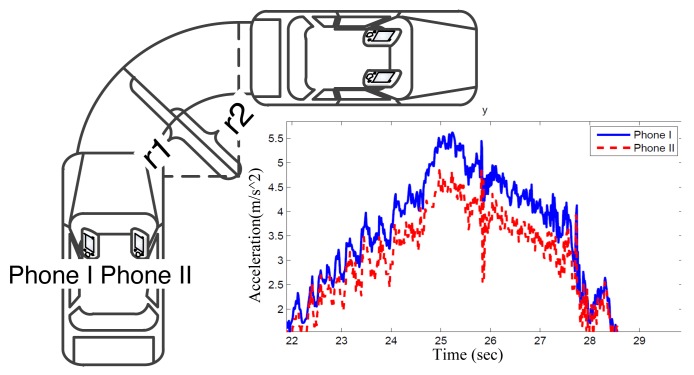
Centripetal acceleration when the vehicle turns.

To distinguish which phone is to the left/right of another in a vehicle, we utilize the difference in the phones' centripetal accelerations measured when the vehicle is turning. Figure 4 illustrates the accelerations of two phones in a car making a right turn. The movement of the car can be approximately regarded as a circular motion. Therefore, both of the mobile phones can record a centripetal acceleration, represented as *a_c_* = *rω*^2^, where *r* is the radius and *ω* is angular velocity. Since both phones have the same angular velocity *ω*, but different radius *r*, the difference in *a_c_* can be used to distinguish the side of the phone. When a car turns clockwise, the *a_c_* recorded at the left phones (Phone I in Figure 4) should have a larger amplitude than Phone II. Similarly, when the car turns counter-clockwise, Phone II should have a larger *a_c_*. Therefore, the objective is to compare two centripetal acceleration time series sampled from two phones to identify which one is generally higher than the other.

Based on our observation at a road intersection, vehicles take 3 s on average to make a 90° turn, which means the mean angular velocity is π/6. Assume that the distance between left and right phones are half of the vehicle width (approximately 0.9 m); we can calculate the difference in accelerations by Δ*a_c_* = Δ*rω*^2^ ≈ 0.9 × (π/6)^2^ = 0.25 *m/s*^2^. As depicted in Figure 4, the centripetal acceleration when the vehicle takes turns varies from 2 *m*/*s*^2^ to 5 *m*/*s*^2^; the difference of 0.25 is significant enough to distinguish them from each other.

**Figure 5.**
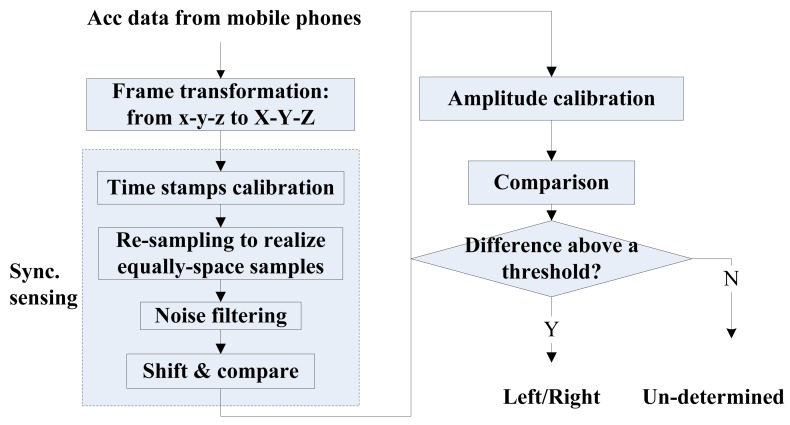
The flowchart of left/right identification.

However, a direct comparison of two centripetal acceleration values, each sampled from a phone, is not able to make a reliable left/right identification, because vehicle speed is not a constant while turning and the measured acceleration data is always noisy. To tackle these challenges, we designed a novel algorithm, and Figure 5 summarizes the procedures we used for left/right identification: after we obtain the centripetal accelerations from different mobile phones, a series of techniques are carried out to ensure these accelerations are synchronized. Amplitude calibration is then carried out to handle the possible differences in mobile phone accelerometers. After that, the output can be compared to determine the left/right position.

### 6.1. Synchronized Sensing

**Figure 6.**
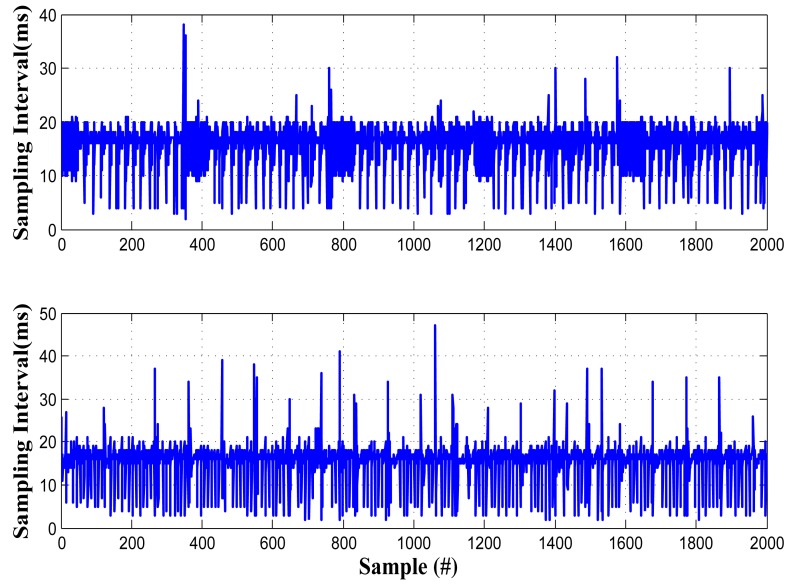
Actual intervals of consecutive samples at two phones (nominal 10 ms).

To compare the acceleration time series from different phones, the phones must be strictly synchronized (<100 ms). One of the traditional synchronization approaches is to exchange beacon signals between two phones or between phones and servers, like NTP [17]. However, this approach is not practical in smartphones, due to the uncertain delay of wireless transmission. Our experiment shows the changes in round trip delay (RTD) can reach as high as 300 ms, which will cause significant synchronization errors. Another possible approach is to utilize the time stamp provided by the GPS system. Unfortunately, modern mobile operating systems (e.g., Android, iOS and Windows Phone) do not provide such an API to access the GPS time stamp. In addition, these methods can only synchronize the physical clocks, but synchronized physical clocks do not necessarily mean synchronized data, since the sensors on the phones can also cause uncertain delay. In our experiment, sampling jitters are observed on many phones, and they show different properties (Figure 6). It also should be noted that the clock crystals of different smartphones are not accurate and may differ from the true value by 10% [18], which also contributes to the unsynchronized sensing in different phones.

As shown in the shaded block in Figure 5, we use the following techniques to realize synchronized sensing on different phones: we first calibrate the time stamps of samples on different phones. Then, we use a re-sampling technique to generate a sequence with equally spaced samples. In this way, the noise in the signal is eliminated. We propose a novel “shift-and-compare” technique to realize synchronized sensing.

**Figure 7.**
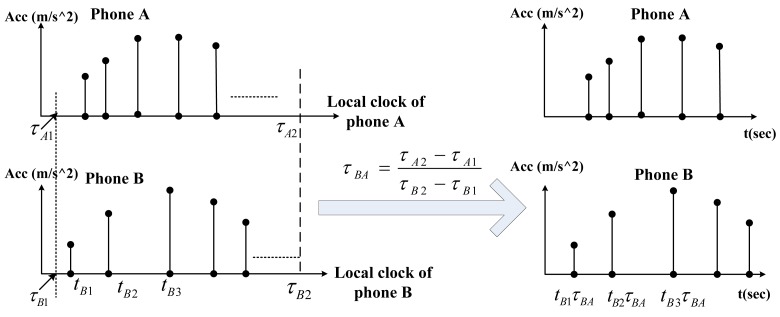
Calibrating the time stamps of Phone B.

**Figure 8.**
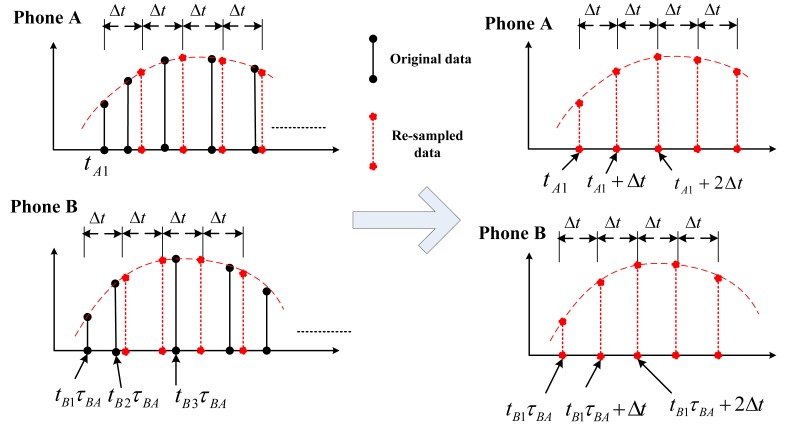
Re-sampling to realize equally-spaced time series.

Time stamp calibration is to guarantee that different acceleration time series have the same clock speed. Assume at its local time *τ_A_*_1_ and *τ_A_*_2_, phone *A* sends a beacon to *B* and later records the local time *τ_B1_* and *τ_B2_* when the corresponding beacon is received. Let *τ_A_*_2_ − *τ_A_*_1_ be large enough, so that the possible delays due to the wireless transmission and the operating system can be ignored. By multiplying $\frac{\tau_{A2}-\tau_{A1}}{\tau_{B2}-\tau_{B1}}$ on each of the time stamps on *B*'s acceleration data, the clock crystals of *A* and *B* are uniformed. This procedure is illustrated in Figure 7, where the time stamps of phone *B* are calibrated. After the time stamp calibration, different phones have the same “clock speed” but the samples may still not be equally spaced in time. We design a re-sampling technique to construct equally-spaced acceleration time series from unequally-spaced data. This technique uses interpolation to estimate values at evenly-spaced time series points [*t*_0_, *t*_0_ + Δ*t*, *t*_0_ + 2Δ*t*, ⋯], where *t*_0_ is the time stamp of the first acceleration point and Δ*t* is the frequency. Note that *t*_0_ is generally different for different phones, but Δ*t* is the same for all phones. This technique is illustrated in Figure 8.

**Figure 9.**
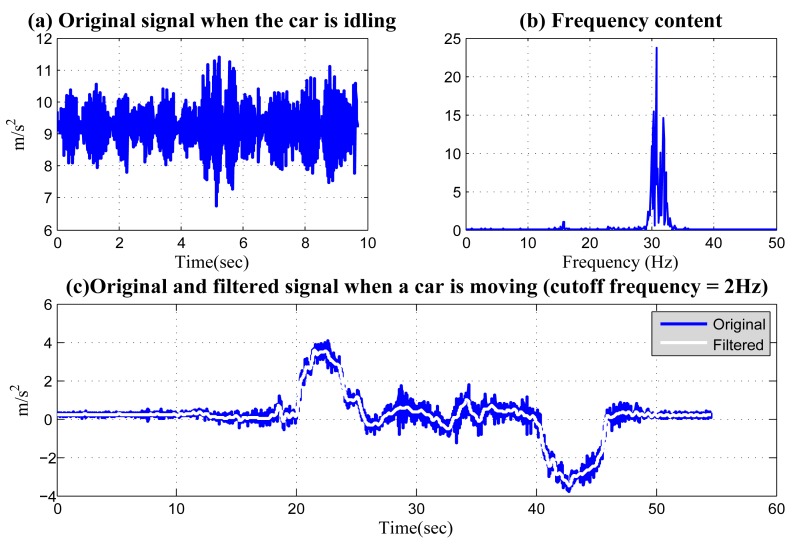
(**a**) The acceleration signal caused by the engine when a vehicle is started, but still idling; (**b**) the frequency content of (**a**); (**c**) the original and the filtered acceleration signal when a vehicle first made a left and a right turn with a cutoff frequency of 2 Hz.

Noise filtering cannot be implemented before the above two procedures, since filtering requires equally-spaced samples with correct time scales. The key of noise filtering is to determine the frequency spectrum of noise and to choose a filter with the correct cutoff frequency to filter out this effectively, while keeping the centripetal acceleration signal unchanged. Figure 9a shows the original acceleration signal measured when a vehicle is idling. These noises are mainly caused by the vibration of the vehicle engine. The frequency spectrum in the signal is shown in Figure 9b. It can be seen that the signal has a large frequency spectrum around 30 Hz. On the other hand, the frequency spectrum of centripetal acceleration used for left/right identification is generally much lower. Figure 9c illustrates the original and the filtered centripetal acceleration signal when a vehicle made two 360-degree turns. The cutoff frequency is 2 Hz. It can be seen that using this cutoff frequency, most of the engine-induced noise is filtered out, while the important features of the accelerations are kept unchanged.

**Figure 10.**
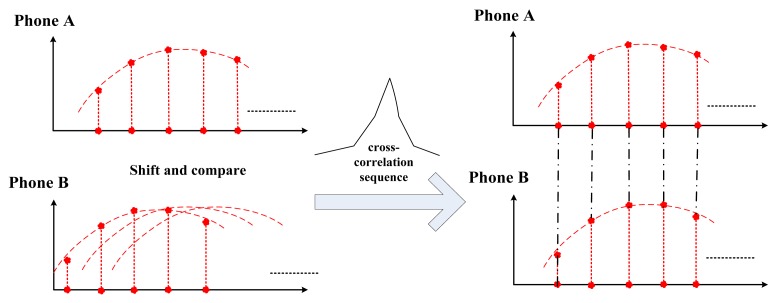
Shift-and-compare to realize synchronized acceleration sequences.

The last step to realize synchronized sensing is to find a starting point for each sequence, such that these starting points correspond to the same global time. This is done by the “shift-and-compare” algorithm. The basic idea is that, for two sequences, *x* and *y*, we fix one *x* and continue to shift *y*, while at the same time calculating the cross-correlation of the two sequences. At the point when the cross-correlation of *x* and the shifted *y* reaches its maximum, the corresponding portions of *x* and *y* are synchronized. This is based on the premise that the acceleration sequences are sampled from phones in the same vehicle and should be similar when these sequences are synchronized. This procedure matches the features contained in the sequences to realize synchronization. This “shift-and-compare” procedure is illustrated in Figure 10.

**Figure 11.**
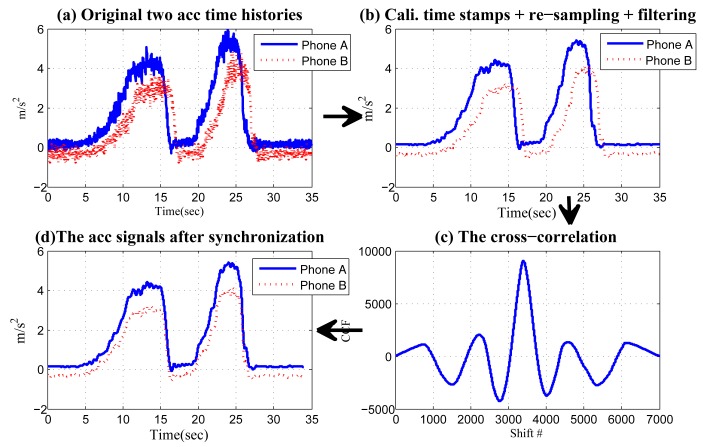
(**a**) Original acceleration time series; (**b**) the series after time stamp calibration, re-sampling and filtering; (**c**) the cross-correlation of the sequences in (b); (**d**) the resultant time series after “shift-and-compare”.

In summary, for synchronized sensing, in Figure 11, we use real data collected in our experiment to demonstrate the entire procedure. The data are collected while a vehicle made two counter-clockwise turns. Notice that when applying “shift-and-compare”, the cross-correlations of the two signals are calculated, and the result is shown in Figure 11c. The location corresponding to the maximum point is 3400, indicating that when the signal from Phone B is right-shifted 3400 times, it has the highest similarity to the reference signal from Phone A. It can be seen from Figure 11d that these two part are strictly synchronized.

### 6.2. Amplitude Calibration

After we obtain the synchronized centripetal acceleration, as shown in Figure 11d, their amplitudes still need to be calibrated before they can be compared. This calibration cannot only eliminate the errors caused by different accelerometers, but also the inaccuracy introduced during reference frame transformation. Note that this calibration is not based on the acceleration signals that are to be compared for left/right identification, but is based on the acceleration data collected when the vehicle is making a translational movement (moving forward). Particularly, the mobile phones will continue to collect data for a period of time (a few seconds) after the turn. Thus, the time series data collected at each phone include two parts: a section collected when the vehicle is turning and another section when the vehicle is moving forward. The data collected during the second section is used to calibrate the amplitude of the first section.

**Figure 12.**
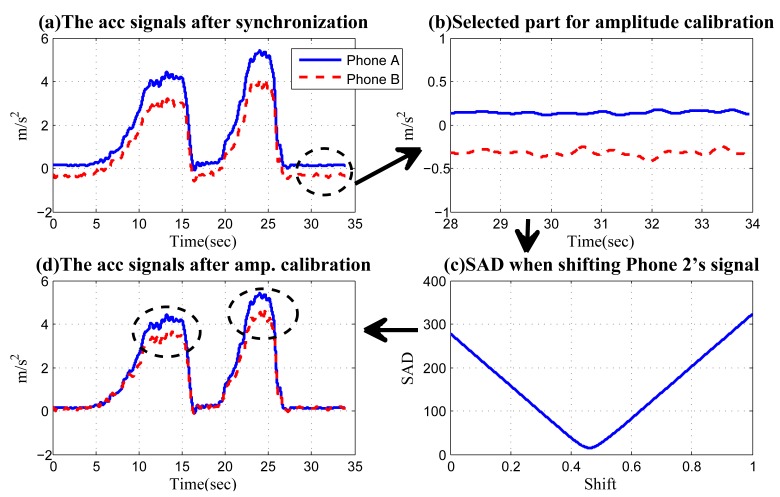
Amplitude calibration.

As an example, Figure 12a shows the synchronized acceleration time series. The corresponding extra sections used for amplitude calibration are circled and shown in Figure 12b. Based on the data in Figure 12b, we use a technique similar to “shift-and-compare”, but the shift occurs vertically instead of horizontally. We select Phone A's signal as the reference time series in Figure 12 and shift the other in a vertical way along the *y*-axis in Figure 12b. When making each shift, the sum of absolute differences (SAD) between the reference time series and the shifted one is calculated, which is shown in Figure 12c. The shift value with the minimum SAD will be used to calibrate the acceleration time series. Figure 12d shows the calibrated acceleration data, and the vehicle turning is marked. Obviously, the difference in the accelerations shows that Phone B has a lower acceleration when the vehicle turns clockwise. Therefore, it is to the right side of Phone A.

## 7. Front/Back Identification

**Figure 13.**
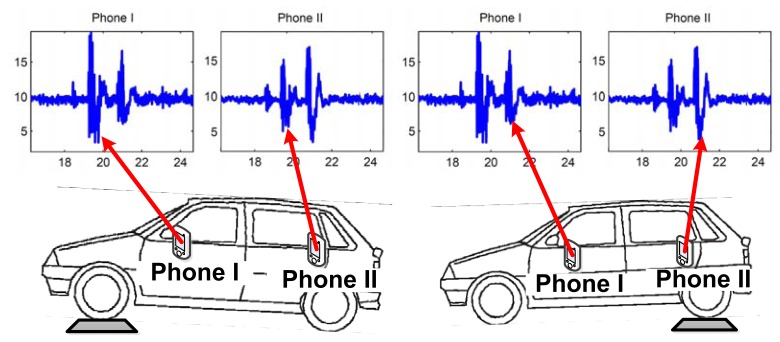
Vertical acceleration when the vehicle passes an uneven surface.

When vehicles pass over uneven road surfaces (e.g., potholes, road connections and bumps), vertical accelerations can be detected by in-vehicle smartphones, which can be used to identify the front/back positions, since the vertical acceleration signals collected from mobile phones at the front seats and the back seats are different. Figure 13 illustrates the phenomenon. Based on the observation, [4] discussed a possible approach to identify two “spike-like acceleration peaks” from the acceleration. If the peak with a larger amplitude is ahead of the smaller one, the phone is located in the front row and *vice versa*.

**Figure 14.**
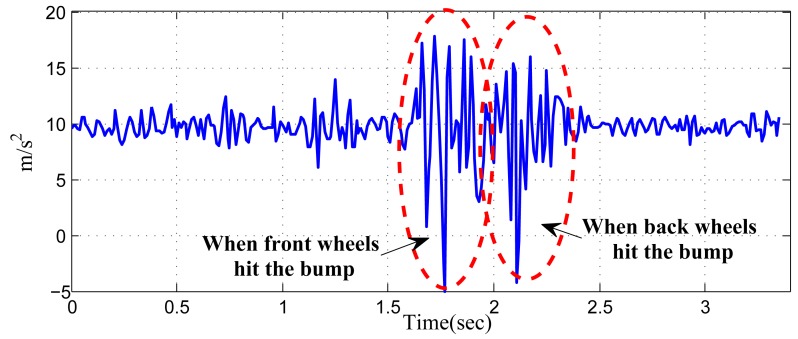
The acceleration data sampled from an in-vehicle phone when a vehicle is passing over a bump and the corresponding two stages.

However, according to our real experiment, such a pattern is not always obvious. This is because when a vehicle passes an uneven surface, the hitting force will generate the vibration of the vehicle, which may last up to a second. As a result, the measured acceleration during this period is highly dynamic. As an example, Figure 14 shows the acceleration data we recorded when a vehicle passes a bump. It can be seen that from a sequence of dynamic data, accurately determining the two peaks, which should correspond to the different stages circled, is difficult, even when noise is not considered.

**Figure 15.**
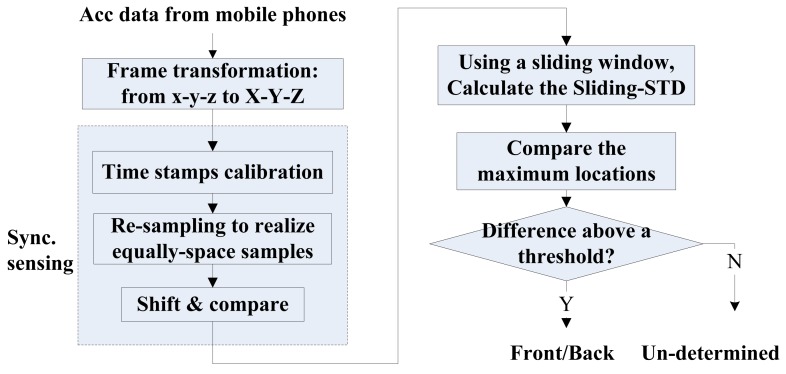
The sliding-SD method proposed to identify front/back locations.

To address this problem, we propose a novel method called sliding-SD. The basic idea is to use a sliding window to calculate the standard deviations (SDs) of acceleration signals in the window. For each phone, we calculate the time point when its SD reaches the maximum. For the two phones, if the difference in the time of maximum SD is above a specific threshold level, the one with an earlier maximum value is in front of another. The procedures in sliding-SD are illustrated in Figure 15. After the collected time series are transformed into vehicle frame, we only take acceleration along the *z*-axis (perpendicular to the vehicle). Notice that compared with the synchronized sensing illustrated in Figure 5, Figure 15 does not contain noise filtering, as the vibration of vehicles is directly utilized for front/back identification and should not be treated as noise. Furthermore, it should be noted that amplitude calibration is not necessary here, since we do not need to compare the signal amplitude from different phones.

**Figure 16.**
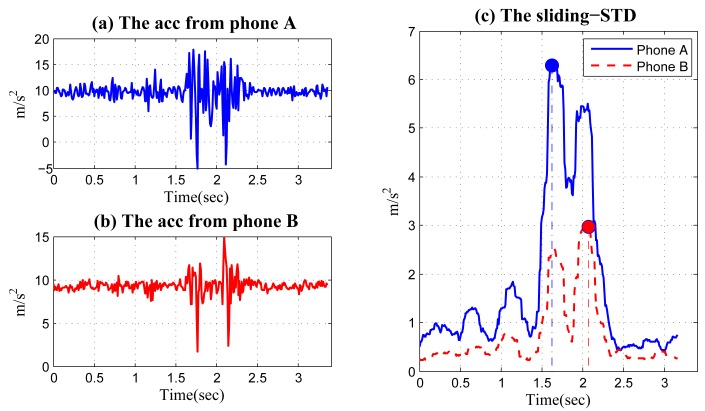
Acceleration signals at the front/back phones when a vehicle passes over a bump: (**a**) when the front wheels hit the bump (**b**) when the back wheels hit the bump.

After the acceleration data from different phones are synchronized, we choose a window with a fixed width, and for each acceleration signal, we shift the window, one point each time, and calculate the SD of the acceleration signal in the window. From the obtained SD sequence of each phone, we identify a maximum value and compare the time when the maximum value occurs. The phone with its maximum SD occurring earlier than another is located at the front seat. This method is illustrated in Figure 16 using data collected in a real experiment. Phone A and Phone B are placed at the front and back seat of a vehicle. Figure 16a,b shows the synchronized acceleration data from the two phones when the vehicle passes a bump. Using a sliding window with a width of 0.3 s, we can obtain two SD sequences in Figure 16c, where the maximum values and the corresponding locations are also shown. It can be seen that the maximum value of the SD sequence of Phone A occurs about 0.45 s ahead of Phone B; then, we can conclude that Phone A is at the front seat and Phone B is at the back.

The proposed sliding-SD method has some significant advantages, compared with finding a higher peak in the two peaks of an acceleration series. First, using SD can capture the vibration change caused by bumps/potholes more accurately than using amplitude directly. Using the SD intrinsically acts as a noise filter. The obtained sliding-SD sequences contain much smaller noise than the original acceleration data, which can be clearly observed in Figure 16c. Second, the sliding-SD method only identifies a single SD peak for each phone, rather than two. This can avoid problems associated with determining the thresholds for the smaller peak and is more robust in the presence of noise.

Finally, we discuss the window width and time threshold. The size of the window should cover the vibration of the vehicle caused by a single hit (front wheels or back wheels). Assume that the wheelbase (the distance between the centers of the front and rear wheels) is *d*, and the speed of a vehicle is *v*; then, the time gap between the two hits is about *d/v*. Therefore, setting a window width to be *d*/2 *v* ∼ *d*/*v* generally works well. In Figure 16c, the window width is 0.3 s, since *d* = 2.7 *m* and *v* ≈ 30 km/h, making *d/v* ≈ 0.32. The discussion above also applies to the time threshold for determining whether a peak in an SD sequence is well ahead of another, such that the front/back location can be assured. Still, take the example in Figure 16c: if we assume the maximum speed of the vehicle when passing bumps and potholes is 60 km/h, then the gap between the SDs from the front and back phones should be larger than *d/v* = 2.7 × 3.6/60 = 0.16 s. If, for example, we find a gap smaller than this threshold, we consider this identification as invalid, and the two phones are perhaps in the same row.

## 8. Experiment and Evaluation

### 8.1. Experimental Methodology

**Figure 17.**
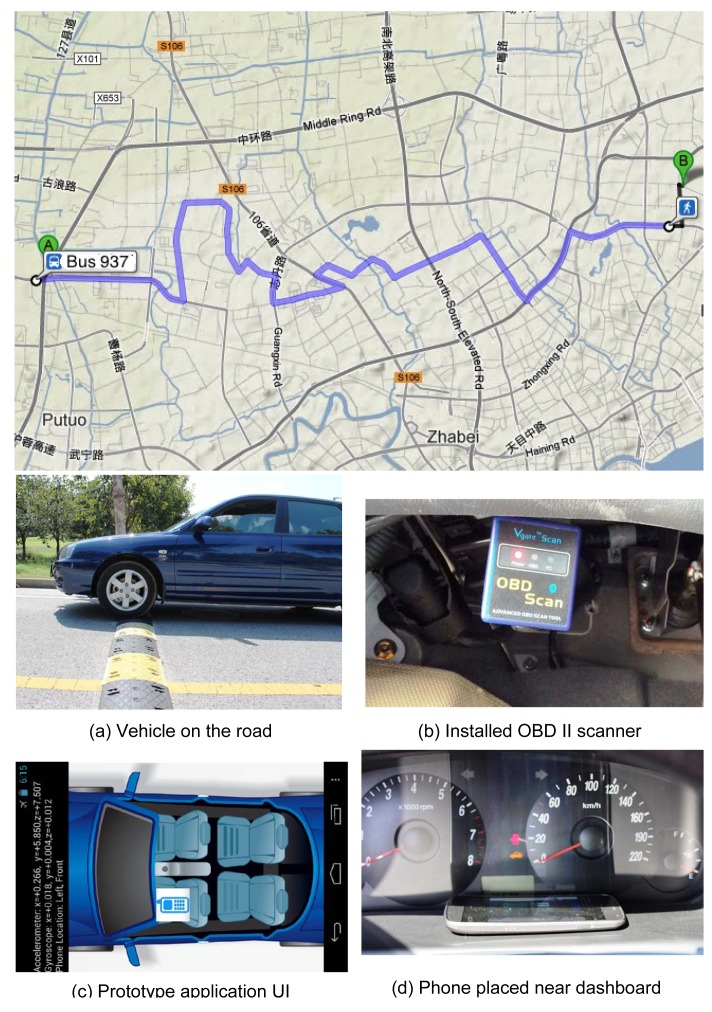
The trajectory of city driving and pictures taken during experiments.

We have implemented iLoc on Android phones with the user interface shown in Figure 17c. Four different Android smartphones are used. Their information can be found in Table 1. All of the phones have accelerometer sensors, but only the Google Nexus series (Phone I and IV) have gyroscope sensors. The system can establish *ad hoc* connections using built-in WiFi programmatically without any manual configuration (like a manual pair-up in Bluetooth). The maximum sample rate for accelerometers we use is 100 Hz. We have tested the solution in a small-sized and a mid-sized sedan, whose parameters are also in Table 1. An OBD scanner is connected to the vehicle to record the speed for evaluation purpose (Figure 17b).

**Figure 18.**
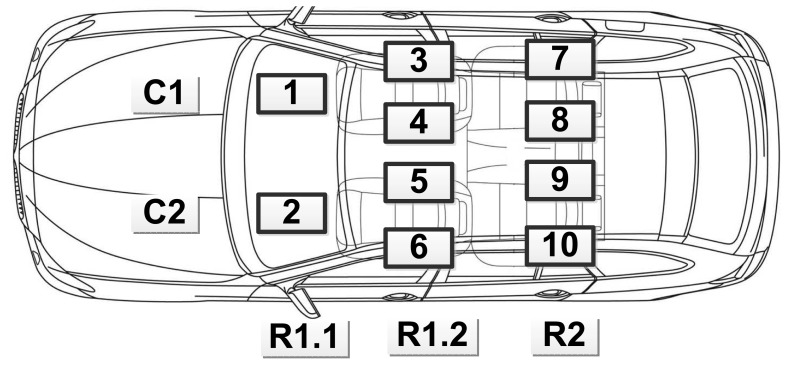
Phone position in the experiment.

Figure 18 illustrates all of the positions of the smartphones that we placed in our evaluation. These positions can be categorized into two rows (R1.1 and R1.2 belong to the front row) and two columns. For Row R1.1, the phones are placed onto the vehicle's center console. For Row R1.2 and R2, the phones are placed inside the driver, or passengers' pocket, or handbag, on different sides. These positions are among the most probable places where a user places their phones inside a car.

We use extensive data collected when driving different vehicles on a 25-km road in Shanghai. Figure 17 shows the driving trajectory that we follow. From the collected data, we found that the 25-km trajectory includes more than 20 turns and more than 50 vertical accelerations caused by uneven road surfaces. Therefore, in a city driving scenario, the events required by iLoc occur quite often. On average, the positioning algorithm can be completed at every kilometer. In order to make our experiment convincing and to help other researchers make further improvements, we have opened the data we collected during our field tests. The data set can be downloaded at our website: http://www.comp.polyu.edu.hk/cszhe/sensordata/.

### 8.2. Left/Right Identification

The accuracy of left/right identification is highly impacted by the acceleration difference Δ*a_c_*. The larger the Δ*a_c_* is, the higher the successful identification probability will be. Therefore, the relative distance between the phones and vehicle speed are the two major factors that influence the accuracy of the algorithm.

To evaluate the impact of relative distance, we fix the vehicle angular velocity by turning at the same road intersection (approximate turning radius: 10 m) with constant speed (about 20 km/h). The phones are placed at Row 1.1 and Row R1.2. For each seat, there are two options. Thus, five combinations can be obtained in total, *i.e.*, {12}, {45}, {35}, {46} and {36}. The impact of angular velocity is evaluated with fixed phone placement (3,5 in Row R1.2). The vehicles make a 90° turn at a road intersection repeatedly at different speeds. The turning period *t* can be obtained by gyroscope readings, and the average angular velocity is then calculated by π/2*t*.

**Figure 19.**
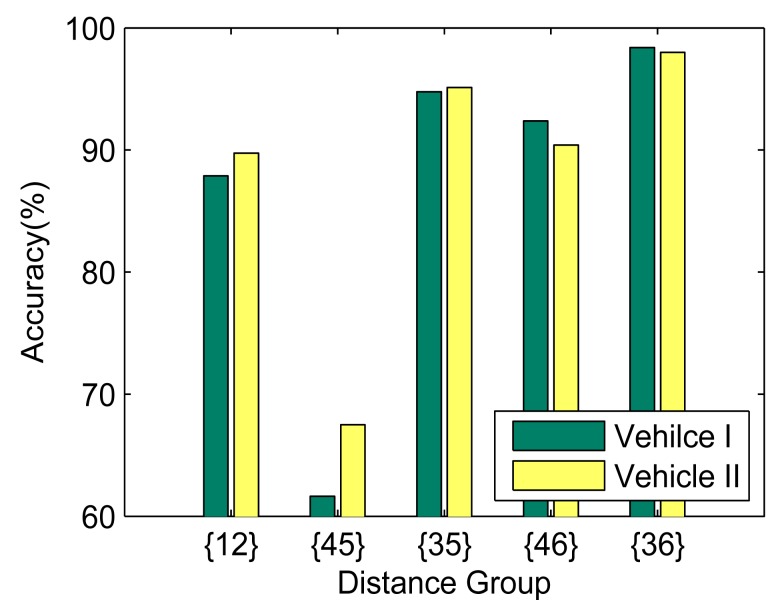
The left/right identification accuracy and the effect of phone distance.

**Figure 20.**
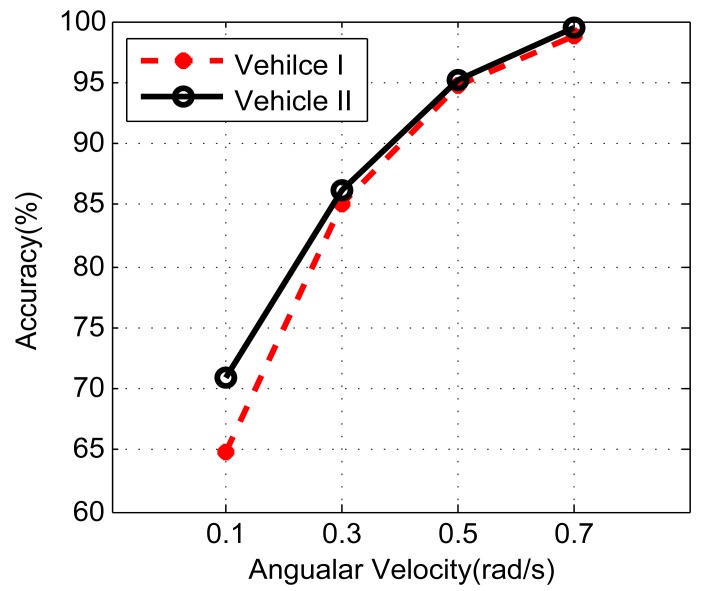
The left/right identification accuracy.

Figures 19 and 20 illustrate the effect of phone distance and vehicle speed on the positioning accuracy, respectively. As the distance between phones grows, the accuracy steadily increases. When phones are placed at {45}, the accuracy is only slightly higher than 60%. However, when phones are placed at {36}, the accuracy is almost 100%. Similar results can also be observed when the vehicle's velocity increases. Under the same configuration, the results from Vehicle II are slightly better than Vehicle I; this is because Vehicle II is about 200 mm wider than Vehicle I, which results in a larger Δ*a_c_*.

**Figure 21.**
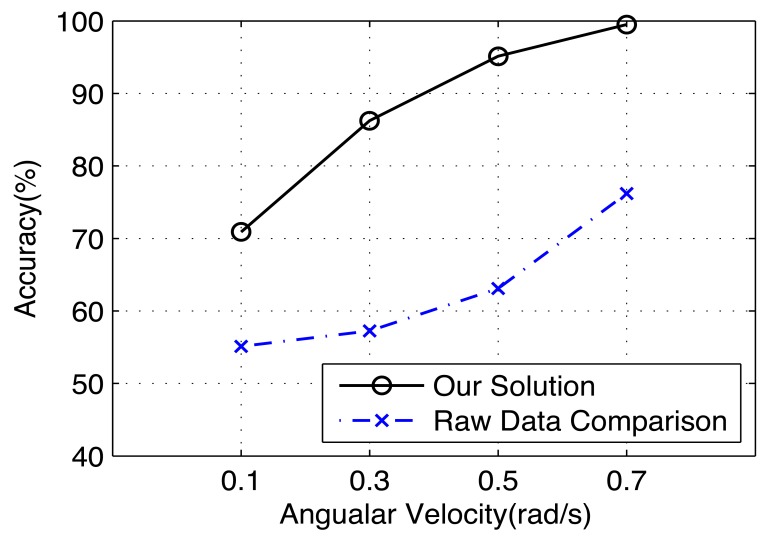
The left/right identification accuracy with/without our proposed data processing algorithm.

Finally, for left/right identification, we evaluate the effectiveness of our proposed algorithm by a direct comparison of the raw sensor data after reference frame transformation. In this experiment, we only use the data from Vehicle II, whose accuracy is generally better than Vehicle I. Figure 21 shows the results. We can see that without using our signal processing algorithms, the accuracy of left/right identification is not satisfactory without using our proposed algorithms.

### 8.3. Front/Back Identification

The accuracy of front/back identification is also affected by relative position and vehicle speed. To evaluate the performance, three phones are placed at Row R1.1, R1.2 and R2. The vehicle passes a bump at different speeds.

**Figure 22.**
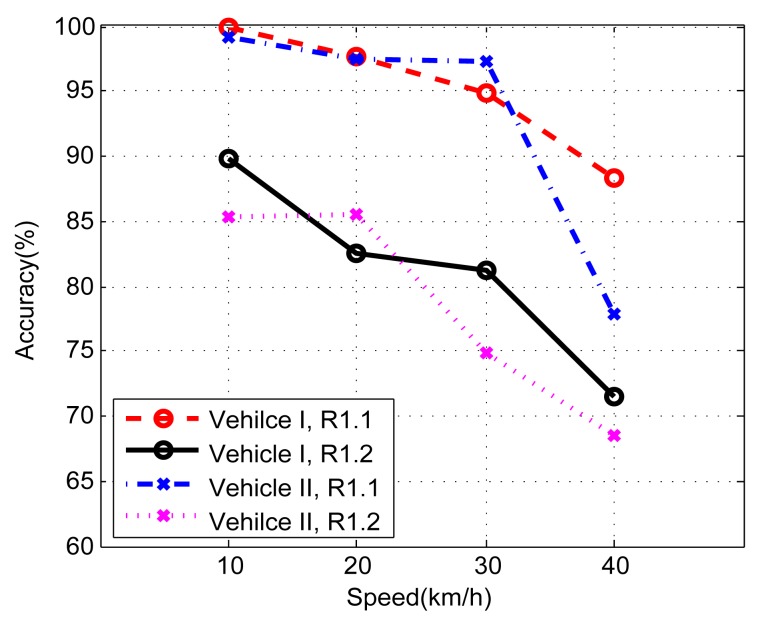
The front/back identification accuracy.

The evaluation results are depicted in Figure 22. The increase of speed results in the decrease of the accuracy, since the time interval between two peaks is smaller. In addition, the accuracy is much lower when phones are placed at Row R1.2; the cause of the result is that Row R1.2 is in the middle of the vehicle, approximately, and the accelerations caused by the two wheels are similar. Under the same configuration, the accuracy of Vehicle I is better than Vehicle II, since the latter has a better vibration damper, which absorbs part of the acceleration along the *z*-axis.

### 8.4. Sliding Window Size in Event Detection

**Figure 23.**
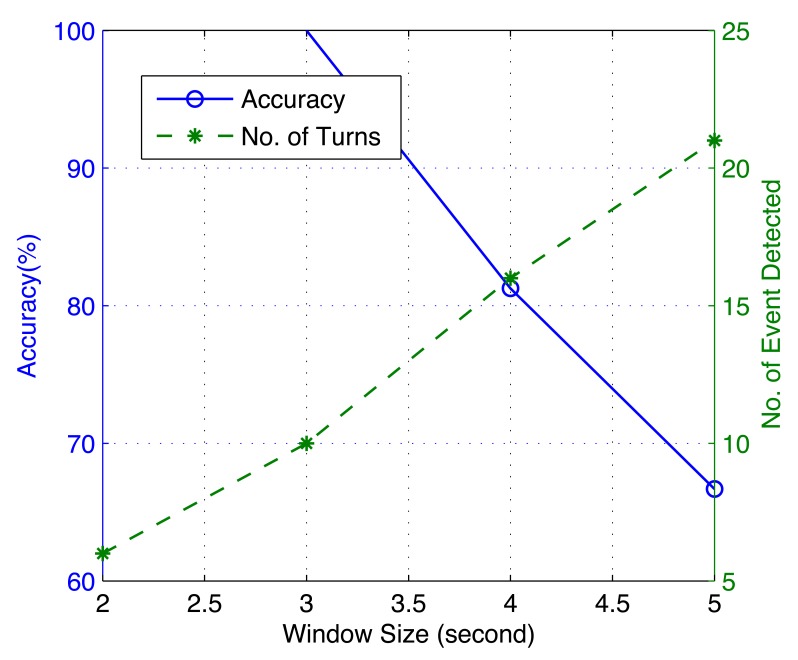
The impact of window size on event detection.

We would like to discuss the effect of the window size used in event detection on the accuracy of iLoc. Take the sliding window size used in vehicle turning detection, for instance: if the window size is large, slow turns whose Δ*a_c_* are not significant enough will be identified as turning events, which decrease the identification rate. On the other hand, a short window size may miss some events, which can result in a longer positioning time. This conclusion is illustrated in Figure 23. It can be seen that when the sliding window size is 5, the accuracy is only 66.67%, but the number of turns that are detected is 21, while when the window size decreases to 2, although 100% accuracy is achieved, only 6 turns are detected, which indicates a long positioning time. In this evaluation, the window size is limited to being larger than 2 s (a vehicle speed of approximately 25 km/h), since it is very dangerous to make turns at a speed larger than 30 km/h.

### 8.5. Energy Efficiency

iLoc mainly consumes battery in three aspects: (1) smartphone sensors for data collection; (2) WiFi for wireless transmission; and (3) the CPU for the execution of the algorithms. With event-triggered sensing, (2) and (3) are only activated when an event occurs. To test the energy consumption, instead of stopping the algorithm after the phones are successfully positioned, we keep it running until the batteries run out. We compare the energy consumption with/without event-triggering. Without event-triggering, iLoc keeps exchanging data between different phones and keeps executing the left/right and front/back identification algorithms. In the city driving experiment, without event-triggering, fully charged Phone I and IV can only last for about 60 min (Phone IV's battery runs out first). However, with event-triggering, after the system runs for 60 min, there is still over 70% battery remaining for both phones.

## 9. Discussion

Through experiments, we noticed that if phones are placed inside users' pockets or held by hand, their movements (adjusting siting position, shaking body) can add additional noise to collected data. These noises are different from those caused by vehicle movement, as there is no correlation among different users' movements. We can utilize this feature to detect and filter these noises in the future.

We also found that iLoc can be improved in the future in the following aspects. Firstly, it is possible that vehicles can run on a long, straight and even highway without any turns or potholes to trigger the sensing. Secondly, iLoc requires multiple smartphones' collaboration to determine their locations. A possible solution to solve the first problem is to utilize the rotation angle or acceleration differences caused by Ackermann steering geometry [19] during lane changes. We have collected some data that already show promising results. For the second problem, we plan to implement a mechanism in iLoc that allows a smartphone to automatically find its location based on a model built from its previously collected data. This model establishes a mapping between its measured data (accelerations, rotation, *etc.*) and its in-vehicle locations under different factors, such as vehicle speed and the radius of turning circle.

## 10. Conclusions

In this work, we proposed iLoc, a seat-level in-vehicle positioning based on smartphone accelerometers. In iLoc, in-vehicle smartphones automatically collect data during certain kinds of circumstances and through cooperation, to determine the relative left/right and front/back seat locations. We have implemented iLoc on Android smartphones and evaluated it in real driving scenarios. The evaluation results show that the accuracy of our solution is promising.

## Figures and Tables

**Table 1.** Phones and vehicles used in the evaluation.

	Phone I	Phone II	Phone III	Phone IV
Model	Galaxy	Galaxy S2	HTC	Nexus S
	Nexus		One V	
CPU Speed	1.2 GHz	1.2 GHz	1 GHz	1 GHz
Accelerometer	3-axis	3-axis	3-axis	3-axis
Gyroscope	3-axis	No	No	3-axis

	**Vehicle I**		**Vehicle II**	

Model	Citroën C2		Hyundai Sonata	
Class	Super mini		Mid-size	
Dimension (mm) (*L* × *W* × *H*)	3665 × 1664 × 1494		4820 × 1835 × 1475	
